# An Integrated Model for Robust Multisensor Data Fusion

**DOI:** 10.3390/s141019669

**Published:** 2014-10-22

**Authors:** Bo Shen, Yun Liu, Jun-Song Fu

**Affiliations:** School of Electronic and Information Engineering, Key Laboratory of Communication and Information Systems, Beijing Municipal Commission of Education, Beijing Jiaotong University, Beijing 100044, China; E-Mails: liuyun@bjtu.edu.cn (Y.L.); 12120067@bjtu.edu.cn (J.-S.F.)

**Keywords:** multisensors, data fusion, Dempster-Shafer theory, extreme learning machine

## Abstract

This paper presents an integrated model aimed at obtaining robust and reliable results in decision level multisensor data fusion applications. The proposed model is based on the connection of Dempster-Shafer evidence theory and an extreme learning machine. It includes three main improvement aspects: a mass constructing algorithm to build reasonable basic belief assignments (BBAs); an evidence synthesis method to get a comprehensive BBA for an information source from several mass functions or experts; and a new way to make high-precision decisions based on an extreme learning machine (ELM). Compared to some universal classification methods, the proposed one can be directly applied in multisensor data fusion applications, but not only for conventional classifications. Experimental results demonstrate that the proposed model is able to yield robust and reliable results in multisensor data fusion problems. In addition, this paper also draws some meaningful conclusions, which have significant implications for future studies.

## 1. Introduction

Multisensor data fusion is a technology to enable combining information from several sensors into a unified result [1]. In multisensor data fusion, the information to be handled is always random, vague, imprecise and heterogeneous. The developed data fusion framework needs to be able to eliminate the redundancy, uncertainty, and fuzziness of the data sources to achieve robust and accurate fusion results. Besides, the framework is required to be able to obtain unified results from multisensor sources, even when conflicts exist among them. Yet data fusion has proved to be valuable in many applications, like pattern recognition and classification [2], image processing [3] and expert systems [4]. Many theories have been applied in multisensor data fusion, such as the Bayesian approach [5], evidential theory [6], fuzzy set theory [7], and rough set theory [8].

The Dempster Shafer evidence theory (DSET), also known as the evidential theory, is a flexible method in multisensor data fusion [9–11]. Its framework is able to deal with information uncertainty, imprecision, randomness, conflicts and heterogeneity. In addition, Dempster's combination rule allows for combining several information sources into a unified one, which makes it popular in multisensor data fusion applications. However, when using DSET in practice, the accuracy of fusion results is always far from satisfactory for two reasons. First, it is difficult to get reasonable and accurate basic belief assignment (BBA); second, it is a thorny problem to make decision with the unified BBA.

A good and effective BBA function, also called mass function, is the prerequisite for applying evidential theory with an aim to combine pieces of evidences into a unified one. There is no general solution for mass function in DSET, and BBAs are usually calculated in heuristic ways. The most typical way is to define the membership functions to mapping data into masses. These methods are easy to implement, such as the fuzzy C-mean algorithm [12], automatic thresholding method [13]. Another way is to develop a transducer mechanism to obtain BBAs by using artificial neural network (ANN) [14–16] and it is has been proved in image data fusion applications. In wireless sensor networks (WSNs), the calculation capacity of sensor nodes is limited, thus implementing the ANN based BBA constructing with high computation complexity is not the most appropriate solution. In DSET, the sources to be combined with Dempster's combinational rule are set to be independent from each other. In reality, it is difficult to meet the condition. Many endeavors have been devoted to solving this problem, and the Belief Transfer Model (TBM) [17] presented by Semts is widely accepted. In TBM, two levels of the beliefs are assumed: the Credal Level (CL) where compound classes are allowed to exist and the Pignistic Level (PL) where beliefs are transferred into probability to make decisions [18]. However, the final pignistic probability is always updated by carving up the intersection classes to the singleton class with a certain proportion. The final decisions are actually determined by the belief of the singleton classes, but have nothing to do with the compound classes [19,20], making the compound classes useless in final decisions. As a result, how to make decisions according to the unified BBA becomes a thorny problem.

To solve the mass constructing problem, a BBA function converting distance to mass is developed in this paper. Distance is a direct and effective reflection measuring the similarity or difference of different classes. It is not affected by data dimension and there are several distance definitions we can choose according to the reality. Besides, distance has a wide range of application, but not only for some specific situations. The key point of the mass function is that the transducer mechanism between distance and BBAs must be reasonable, effective and flexible. A radial basis function can be used to mapping distance to BBAs of different subsets, and the obtained BBAs should be able to adjust according to different parameters. As a result, the mass function will be able to adaptively generate reasonable BBAs according to the given sample sets.

Another endeavor to solve the mass constructing problem is presenting an algorithm to synthetize BBAs from different BBAs obtained by different functions or experts. The reason is that different BBA functions or experts maintain their own positions and we've found that the synthetic BBA is always more reliable than independent BBAs. However, how to build reasonable BBA synthetizing algorithm becomes another problem. The Jousselme distance [21] is a widely accepted way of measuring distance between two evidences bodies and it is able to reflect the conflict degree of evidences properly. Hence we develop the synthetizing algorithm by utilizing Jousselme distance to get synthetic BBAs, which represents a comprehensive knowledge of the information source by combining the views of different BBA functions or experts.

The decision making mechanism is also vital to obtain high accurate fusion results. In the perspective of dimensionality reduction, the unified BBA can be regarded as the comprehensive presentation of the original multisensor data. It's much more easily to make decision with the unified BBA rather than the original data. Traditionally, the Belief Transfer Model (TBM) is always used to convert the final BBA to Pignistic probability. However, the transferred Pignistic probability essentially depends on the singleton classes while the compound classes have no decisive influence on the final decision. In some conditions when the belief assignments of the compound classes are larger than the singleton classes, the accuracy of TBM is suspicious. Thus a decision making mechanism based on Extreme learning machine (ELM) is presented to solve the decision making problem. ELM [22] is a fast and easy implementing ANN without iterated operation, and to our knowledge, the accuracy of ELM is no worse compared to any other ANNs. Thus a decision making mechanism based on ELM is presented to solve the decision making problem.

A systematic multisensor data fusion model is built up in the basis of the above three main improvements. The framework will be illustrated in detailed, which includes four steps: BBA construction, BBA synthesis, combination of evidences and decision making. Experimental results and analysis on the IRIS data set and Diabetes Diagnosis data set will be illustrated to show the performance and result accuracy of the proposed algorithm.

The remainder of this paper is organized as follows: Section 2 introduces the preliminaries of thee Dempster Shafer evidence theory. Section 3 illustrates the proposed method in detailed. The experiments along with the observations are provided in Section 4. Conclusions and discussions are finally presented in Section 5.

## 2. Preliminaries of Dempster Shafer Evidence Theory

Dempster Shafer evidence theory (DSET) is an extension of the classical probability theory. It is a flexible evidential reasoning approach for dealing with the uncertainty in multisensor data fusion. Let Ω = (*ω*_1_,…,*ω_c_*) be a finite non-empty set and Ω is mutually exclusive and exhaustive. Ω is called the frame of discernment, the corresponding power set is , which is composed by all possible subsets of Ω. The mass function of 2^Ω^ is defined as a function m: 2^Ω^ → [0, 1] and it satisfies the following property:

(1) $$\begin{array}{lll} \sum_{A\subseteq \Omega }m(A)=1 & \text{and} & m(\emptyset ) \end{array}=0$$

where Ø denotes the null set, *m*(*A*) is called the basic belief assignment (BBA) of subset *A*. The numerical value of *m*(*A*) can be interpreted as the support degree of proportion *A* belongs to Ω with all relevant and available evidences. A focal element is a subset *A* with non-zero mass assignment and we call (*A, m*(*A*)) a piece of evidence.

For a given element *A* of Ω, the belief function and plausibility function of *A* are denoted by *Bel*(*A*) and *Pls*(*A*), respectively. *Bel*(*A*) is the total mass of elements belonging to *A* and *Pls*(*A*) is the maximum total mass of elements that may be distributed in *A*. Therefore, they are defined as the following expressions:

(2) $$\text{Bel}(A)=\sum_{B\subseteq A}m(B)$$

(3) $$\text{Pls}(A)=\sum_{A\cap B\neq \emptyset }m(B)$$

where *Bel*(*A*) and *Pls*(*A*) are the lower and upper belief of hypothesis *A*, respectively. *Bel*(*A*) and *Pls*(*A*) satisfy the following relation:

(4) $$\text{Pls}(A)=1-\text{Bel}(\bar{A})$$

(5) Pls(A)≥Bel(A)

where *Ā* is the complementary set of *A*. When *Pls*(*A*) = *Bel*(*A*), *A* must be a singleton class. For an arbitrary focal element in Ω, its BBA distributes in an explicit measure of a belief interval [*Pls*(*A*), *Bel*(*A*)].

In DSET, The Dempster combinational rule can be used to fuse all independent evidences into one. It is expressed as:

(6) $${⊕}_{i=1}^{n}m_{i}\left(A\right)=\frac{1}{1-K}\sum_{\cap_{i=1}^{n}A_{n}=A}\prod_{i=1}^{n}m_{i}\left(A^{i}\right)$$

(7) $$\text{with}\;K=\sum_{\cap_{i=1}^{n}A_{n}=\emptyset }\prod_{i=1}^{n}m_{i}\left(A^{i}\right)$$

where ⊕ denotes the combinational operator. *A_i_* designates the focal element regarding to data source *i*. *K* indicates the conflict among the sources to be combined. After combining, a Pignistic probability can be obtained by using the Transfer Belief Model (TBM) and a typical transfer formula is defined as [17]:

(8) $$\text{Bet}P(A_{i})=\sum_{A_{l}\subseteq A_{M}}\left(\frac{1}{\left|A_{M}\right|}\right)m(A_{M})$$

where Bet*P*(*A_i_*) is the transferred Pignistic probability regarding to *A_i_*. At last, a decision can be made by choosing the class with maximum Pignistic probability as the result of the multisensor data fusion process.

## 3. The DSET-E Multisensor Data Fusion Model

### 3.1. Overview

**Figure 1.**
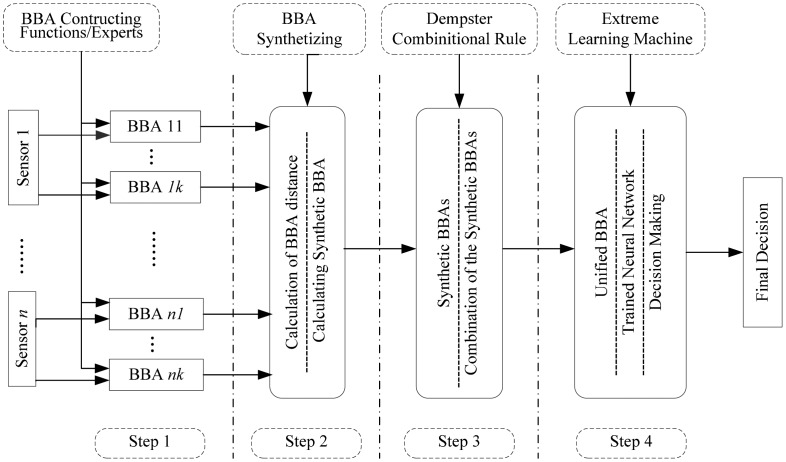
Process of the proposed evidential multisensor data fusion algorithm.

The main goal of the framework is to guarantee accurate and robust decision-level fusion results, even in situations with high complex and nonlinear data sources. Thus, robustness is the principle of the algorithms in the entire process and it is guaranteed by three aforementioned reliable and robust measures: BBA constructing, BBA synthetizing and decision making. The process of the data fusion model is divided into four steps, as shown in Figure 1.

Let Ω = (*ω*_1_,…,*ω_c_*) be the frame of discernment containing *c* elements, *F* = {*F*_1_,…,*F_k_*} are the BBA functions or experts, **S** = {*s*_1_,…,*s_n_*}are a set of sensors. In step 1, there are *k* functions or experts and *n* sensors. Every BBA constructing function/expert will generate a BBA regarding to a sensor, hence there are *n* × *k* BBAs obtained in this step 1. Step 2 is the BBA synthesis process involving a developed synthetizing algorithm, based on which the *n* × *k* BBAs will be decreased into *n* synthetic BBAs. The combination calculation is conducted in step 3. Using the Dempster's combinational rule, the *n* synthetic BBAs will be combined into one unified BBA. Step 4 is the decision making process. With a trained ELM, the unified BBA will be transferred into the final output, which is easy for making decisions.

### 3.2. BBA Constructing Function Model

For decision fusion, local classification or decision results are essential before fusing them into a unified one. In scenarios when there is a large amount of raw data, directly uploading them to the cluster node (or sink node) is very costly. However, uploading local decision results will greatly reduce the amount of data transmission, and greatly reduce energy consumption, which is significant for distributed sensor networks, especially wireless sensor networks (WSNs). Therefore, developing a BBA constructing algorithm is necessary to obtain the local classification results.

In expert systems, BBAs are constructed based on human decisions. This paper focuses on constructing a BBA from data sources. Distance is a widely used metric measure of the similarity of an object and a class. However, each kind of distance maintains its own views. Let *S* be the object to be classified. The training set is Γ = {(*φ*_1_, *ω*_1_), ⋯ , (*φ_c_*, *ω_c_*)} and *φ_i_* (*i* = 1,..,*c*) is the training class respect to *ω_i_*. There are various kinds of distances, such as Euclidean distance, Mahalanobis distance, Manhattan distance, to name a few. Here, we set *k* distance expressed as = {*dis*_1_,…,*dis_k_*}, where *dis*_ν_ (ν = 1, …, *k*) is the νth distance definition of *D*.

Firstly, the distance between an object and each sample class must be calculated. The general distance can be expressed by the following expression:

(9) $$d_{\nu i}=dis_{\nu }(\left\|s-\phi_{i}\right\|),\nu =1,...,k,i=1,...,c$$

where *d_υi_* is the distance of *S* and *φ_i_* according to the *ν*th distance definition in *D*. Sample set *φ_i_* can be the whole given sample set or the nearest *m* objects around the object in the given sampe set. If an object belongs to a class *ω_i_*, then the mass should be assigned to two subsets of Ω, and they are {*ω_i_*} and Ω. Then the assigned BBA *m_ν_*(·|*φ_i_*) can be defined as:

(10) $$m_{\nu }(w_{i}|\phi_{i})=\alpha f_{\upsilon }(d_{\upsilon i}),0<\alpha <1$$

(11) $$m_{\nu }(\Omega |\phi_{i})=1-\alpha f_{\nu }(d_{\nu i})$$

(12) $$m_{\nu }(B|\phi_{i})=0\forall B\subset 2^{\Omega }\backslash \{\{\omega_{i}\},\Omega \}$$

where *m*_ν_ is the assigned BBA of *S* according to the *v*th distance definition in *D*. α ∊ (0, 1) is a positive constant and *f_ν_* is a monotonically radial basic function decreasing form *f_ν_*(0) = 1 to $\underset{d\to \infty }{\lim}f_{\nu }(d)=0$. It can be postulated as:

(13) $$f_{\nu }(d_{i})=\exp(-r_{\nu }{\left(d_{\upsilon i}\right)}^{2})$$

where *m_ν_* is a positive constant value. In [23], a method for optimizing parameters *α* and *γ* has been described by using of KNN method. Here we give a more exact form of γ and it is defined as:

(14) $$\gamma_{\nu }=\frac{1}{{\bar{d}}_{\nu }^{2}}\ln2$$

(15) $$\begin{array}{ll} \text{with} & {\bar{d}}_{\nu }=\frac{1}{c}\sum_{i=1}^{c}d_{\nu i} \end{array}$$

where *d̄ν* is the mean value of distance between the object and each class in Ω. *α* can be changed to adjust the discrimination degree of the obtained BBA. After considering each pattern, the BBA function ν to be calculated as:

(16) $$m_{\nu }=m_{\nu }(\cdot |\phi_{1})⊕\cdots ⊕m_{\nu }(\cdot \phi_{c})$$

where *m_ν_* is the BBA with respect to function *F_ν_*, *m_ν_*(·*φ_c_*) is the BBA calculated by Equations (10)–(12). With the *k* different BBA functions, for a hypothesis *A*, the generated corresponding BBAs are {*A|m*_1_(*A*),…,*m_k_*(*A*)}. Next step involves composing the synthetic BBA from the *k* BBAs generated by different functions.

### 3.3. BBA Synthetic Algorithm

Correct BBAs are the prerequisite for applying DSET in multi-source information fusion. In reality, there is no universal applicable BBA-constructing function. Different kinds of BBA functions are based on different theories, and each of them maintains its own views. For one information source, BBAs generated by different functions are always different. Dempster's combinational rule requires mutually independent evidences, and it cannot be applied to combining BBAs obtained from the same source. Hence, a new method for combining these BBAs into a comprehensive one that achieves a wide range of aspects of uncertainty is significant for obtaining reasonable synthetic BBAs. According to reference [21], the distance of evidence can be defined as:

(17) $$d_{BBA}(m_{1},m_{2})=\sqrt{\frac{1}{2}{\left(\vec{m_{1}}-\vec{m_{2}}\right)}^{T}\underset{͇}{D}\left(\vec{m_{1}}-\vec{m_{2}}\right)}$$

where *m⃗*_1_ and *m⃗*_2_ are evidence vectors. *D* is a positively defined matrix and its coefficients can be obtained as:

(18) $$D(A_{1},A_{2})=\frac{\left|A_{1}\cap A_{2}\right|}{\left|A_{1}\cup A_{2}\right|}$$

In order to transform the reliability of a function to metric, we define the credibility of a BBA as:

(19) $$a_{\nu }=1-\frac{\sum_{j=1}^{k}d_{BBA}(m_{\nu },m_{j})}{\sum_{j=1}^{k}\sum_{j=1}^{k}d_{BBA}(m_{i},m_{j})}$$

where *a*_ν_ is the credibility of BBA with respect to mass function *m_ν_*. The expression above means that a BBA with further distance will be assigned with a low credibility value and compared with other BBAs, and it is not reliable. The sum of credibility is:

(20) $$\text{sum}(a_{\nu })=\sum_{\nu =1}^{k}\left(1-\frac{\sum_{j=1}^{k}d_{BBA}(m_{\nu },m_{j})}{\sum_{i=1}^{k}\sum_{j=1}^{k}d_{BBA}(m_{\nu },m_{j})}\right)=k-1$$

Thus, the normalized a*¯_i_* can be calculated by:

(21) $$\bar{a_{\nu }}=\frac{1}{k-1}a_{\nu }=\frac{1}{k-1}[1-\frac{\sum_{j=1}^{k}d_{BBA}(m_{\nu },m_{j})}{\sum_{i=1}^{k}\sum_{j=1}^{k}d_{BBA}(m_{i},m_{j})}]$$

The sum of a*¯_i_* is $\sum_{i=1}^{k}{\bar{a}}_{i}=1$ . Then, the synthetic BBA can be calculated by the following weighted sum method:

(22) $$m(A)=\sum_{\nu =1}^{k}\bar{a_{\nu }}m_{\nu }(A),\nu =1,...k$$

where *m*(*A*) denotes the hybrid BBA with respect to hypothesis *A*, *ā_i_* is the weight of BBA with respect to function *F_i_*. The value *ā_i_* shows the reliability of the corresponding function. A believable function will be assigned a larger credibility compared to other functions. The final obtained BBA will be regarded as the local decision and uploaded to the fusion center.

### 3.4. Combination of the Synthetic BBAs

The Dempster's combinational rule has been widely accepted as a method for combining various evidences into a unified one. With the obtained *n* evidences, the unified evidence can be defined as:

(23) $$(m_{1}⊕\cdots ⊕m_{n})(A)=\frac{1}{1-k}\sum_{A_{1}\cap \cdots \cap A_{n}=\emptyset }m(A_{1})\cdots m(A_{n})$$

(24) $$\begin{array}{ll} \text{with} & K= \end{array}\sum_{A_{1}\cap \cdots \cap A_{n}=\emptyset }m(A_{1})\cdots m(A_{n})$$

where *K* denotes the conflict degree, final unified BBA contains the comprehensive knowledge of all information sources. Note that other combinational rules, including the method proposed in Section 3.3., can be applied instead of the Dempster's combinational rule.

### 3.5. ELM Based Decision Making Model

Unlike the traditional TBM transfer mechanism, this paper uses a decision-making algorithm based on ELM. Huang and his colleagues [24] proposed extreme Learning Machine (ELM). This single feed-forward neural network (SLFN) with a fast learning speed has both universal approximation and classification capabilities [25]. In ELM, the input weights of the hidden neurons can be generated randomly, and they are independent from applications. Thus, ELM requires no iterative calculation to determine the input weights, which is a big advantage of the traditional artificial neural networks.

**Figure 2.**
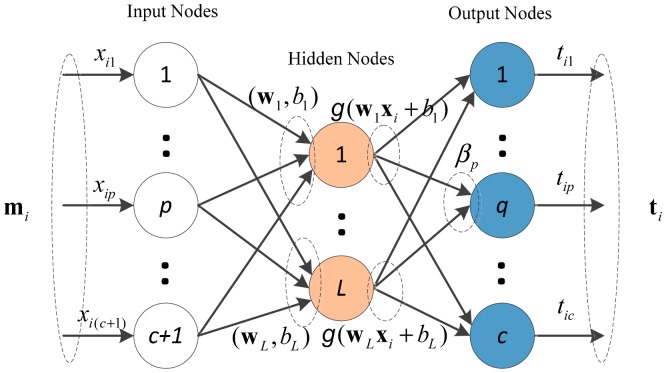
Decision making network of ELM.

As shown in Figure 2, ELM can be directly applied to conduct the decision making process. For *N* arbitrary distinct samples (**m**_1_, **t**_1_) (*i* = 1,…, *N*), where input data **m***_i_*= (*m_i_*(*ω*_1_),…, *m_i_*(*ω_c_*), *m_i_*(*ω*_Ω_)) ∊ *R^c^*^+1^ and output data **t***_i_* = (*t_i_*_1_, … , *t_ic_*) ∊ *R^c^* the output of the network with *L* hidden neural nodes can be expressed as:

(25) $$t=\sum_{j=1}^{L}\beta_{j}g(w_{j}m_{i}+b_{j}),i=1,...N$$

where **t***_i_* is the network output of **X***_i_*, **w***_j_* = [*w_j_*_1_,.. *w_j_*_(c+1)_]*^T^* is the input weight matrix between the input neural node and the *j*th hidden neural nodes, *β_j_* = [*β_j_*_1_, … ,*β_jc_*]*^T^* is the output weight matrix between the *i*th hidden nodes and output nodes, *b_j_* is the bias threshold respect to the *i*th hidden node. **w***_j_* and *b_j_* are generated randomly and are independent from any specific applications.

Let *g*(*x*) be the activation function. The above *N* equations can be written as:

(26) Hβ=T

with:

(27) $$\text{H}={\left[\begin{array}{ccc} g\left({\text{w}}_{1}{\text{x}}_{1}+b_{1}\right) & \cdots & g\left({\text{w}}_{L}{\text{x}}_{1}b_{L}\right)\\ \vdots & \cdots & \vdots \\ g\left({\text{w}}_{1}{\text{x}}_{N}+b_{1}\right) & \cdots & g\left({\text{w}}_{L}{\text{x}}_{N}b_{L}\right) \end{array}\right]}_{N\times L},\beta {\left[\begin{array}{c} \beta_{1}^{T}\\ \vdots \\ \beta_{2}^{T} \end{array}\right]}_{L\times m}\mathrm{and}T={\left[\begin{array}{c} {\text{t}}_{1}^{T}\\ \vdots \\ {\text{t}}_{N}^{T} \end{array}\right]}_{N\times m}$$

where **H** is hidden neural output matrix. The output matrix *β* can be calculate by:

(28) $$\beta ={\text{H}}^{†}\text{T}$$

where **H**^†^ is the Moore–Penrose generalized inverse of matrix **H**. To improve the robustness of the generalization performance, the above expression can modified as [22]:

(29) $$\beta ={\left(\frac{\text{I}}{C}+{\text{H}}^{T}H\right)}^{-1}{\text{H}}^{T}\text{T}$$

The ELM algorithm can be designed in 3 steps: (1) Assign the input weight matrix **W** and bias *b* randomly; (2) Calculate the output matrix **H** of the hidden neural nodes; (3) Calculate the output weight matrix *β*. After training the ELM, it is able to perform specific functions, like approximation and regression.

Given *N_o_* new observed unified BBAs with masses **m***_o_* ={*m*_o1_, … ,*m_oNo_*} and the respective *m-*th one is **m***_om_*, = *m* = 1,…,*N_o_*, , the output of the ELM is:

(30) $${\text{t}}_{o}=g\left(\text{w}\cdot {\text{m}}_{o}+b\right)\beta$$

where **t**_o_ is the output matrix and **t**_o_ = [**t**_o1_,…, **t**_o2_] Then the decision can be made by:

(31) $$\omega_{Dg}=\max\left({\text{t}}_{og}\right),g=1,\ldots ,c$$

where *ω*_Dg_ is the final decision with respect to *g-*th column in **t**_o_. Note that other ANN algorithms, like BP and RBF, also can be applied in this step, the decision making policy is the same as ELM.

## 4. Experimental Results

This section reviews the experiments that are performed to test the performances of the proposed data fusion algorithm. In the foregoing experiments, we simulate our model in three steps: First, we use the IRIS dataset to illustrate the performance of the proposed mass construction algorithm. Second, we use the Diabetes Database dataset to train our model, and then, we collect data from the people whose age range from 40 to 60 by human body sensors, and predict their health condition. In addition, the last experiment applied the proposed framework in vehicle type classification. Introductions about these tests and their corresponding results will be described in the following sections.

### 4.1. Experiment on IRIS Data Set

In this experiment, we use the IRIS data collected by statistician Fisher [26] to simulate the algorithm. There are three species of Iris in this data set: Setosa (Se), Versicolor (Ve), and Virginica (Vi), and each plant includes four indices: sepal length (SL), sepal width (SW), petal length (PL), and petal width (PW). The total number of data is 600 (150 × 4), or 50 patterns for each plant. To classify the plants, each of the four features represents an information source. Among the 50 patterns of each plant, 30 patterns are randomly selected as the training sets, the remaining 20 patterns of each plant are the testing sets.

**Figure 3.**
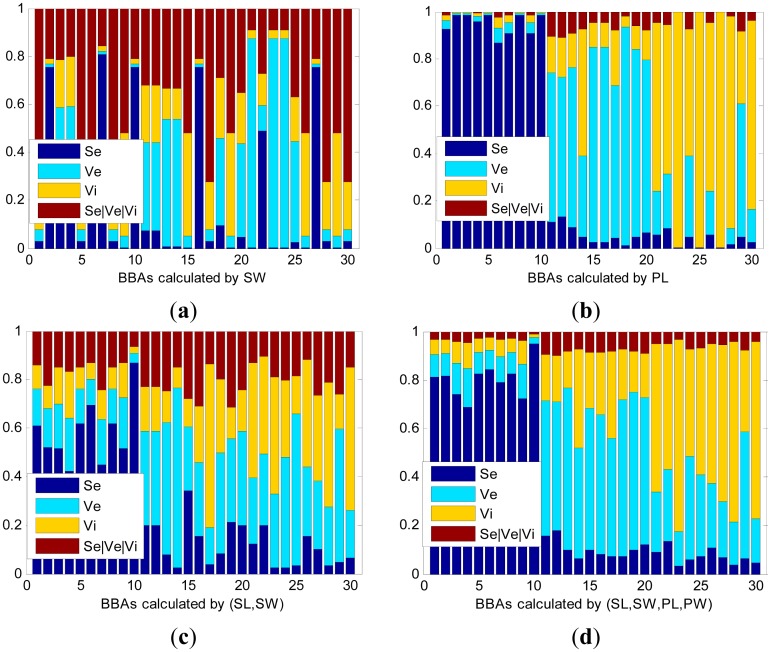
Partial BBAs calculate by the Eu-function includes (**a**): training set SW; (**b**): training set PL; (**c**) training set (SL, SW); (**d**) training set (SL, SW, PL, PW).

Four kinds of distances are used in the experiment: Euclidean Distance (Eu), Mahalanobis Distance (Mahal), Chebyshev distance (Ch), and Manhattan Distance (Ma). Different functions generate BBAs with different accuracies. The test data sets include seven patterns, which are SL, SW, PL, PW, (SL, SW), (PL, PW), and (SL, SW, PL, PW). The BBAs are calculated using Eu, as shown in Figure 3.

After obtaining all BBAs from different dimensional data set and BBA functions, the synthesized BBAs of the four distance BBA functions can be obtained. To examine the rationality and validity of the proposed functions, the accuracies are calculated in every step of the BBA constructing process. The accuracy degree of a BBA is calculated using the following expression:

(32) $$r\left(\omega_{i}\right)=\frac{N_{y}\left(m\left(\omega_{i}\right)=\max\left\{m\left(\omega_{1}\right),\ldots ,m\left(\omega_{c}\right)\right\}\right)}{N}\times 100\%,i=1,\ldots ,c$$

where *r*(ω*_i_*) denotes the accuracy rate with respect to class *ω_i_*,*N N* is the total BBA number of the test sets. *N_y_* is the number of accurate BBAs. If the object to be classified belongs to *ω_i_*, *m*(*ω_i_*) should be larger compared to any other singleton classes. The accuracies of the results are calculated by the 7 source data set. The accuracies of the BBAs for different sources and BBA functions are shown in Table 1.

Figure 3 shows partial BBAs calculated using the Euclidean Distance. The horizontal axis denotes the number of test objects and vertical axis represents the mass assignments of each object. The sum of each mass equals 1. The first 10 objects are Se, next 10 objects are Ve, and the last 10 objects are Vi. In Table 1, the syn-BBA denotes the BBAs synthesized from the four BBAs with different distance definitions. We make the following observations:

(1) The proposed mass construction method is able to build BBAs from observed data and information accurately and effectively. With a distinguishable data set, the mass of the compound classes will be much lower compared to the sum of the singleton classes, and the belief assignment with respect to the class to which the object belongs will always be much larger compared to other classes. As shown in (b), (c), (d) and Table 1, the accuracies of PL, (SL, SW), and (SL, SW, PL, PW) calculated by the Eu are 96.67%, 83.33%, and 98.33%, respectively. While given an ambiguous data set, as shown in (a), the masses of each object will likely to be confusing, significantly decreasing the accuracy rate and belief assignments of _Ω_ (Se|Ve|Vi), with the accuracy being only 58.33%.
(2) Higher dimensionality data enhances the accuracy of BBAs. With the same BBA function in (a), the BBAs obtained from SW have low accuracy (58.3%), while the accuracy is much higher in (c) (83.33), where the BBAs are calculated by (SL, SW). In (d), the BBAs’ accuracy is 98.33% and the dimension is 4. In a more dimensional space, the boundaries of the plants can be classified more clearly. Generally, higher dimensional data brings more accurate BBAs, as the BBAs accuracies of (SL, SW, PL, PW) are higher compared to others, except for the Ch-BBA function.
(3) The synthesized BBA is able to comprehensively illustrate the data source. When assigning belief for an information source, different methods hold their own views and their results may different, too. Thus, the method of synthesizing different BBAs into a unified comprehensive BBA is able to get that the result reflecting the views of the majority.

### 4.2. Experiment on Diabetes Data Set

The Pima Indians Diabetes Database (available at [27]) was developed at the Applied Physics Laboratory, Johns Hopkins University. The eight indices in the data represent the diagnostic signs of diabetes according to World Health Organization criteria. The database comprises the data from 768 women over the age of 21 residing in Phoenix (Arizona, USA). All examples belongs to either positive (denotes by 1) or negative (denotes by 0) class. All input values are within [0, 1]. To test the proposed method, 75% (576) and 25% (192) samples are chosen randomly for training and testing at each trial, respectively.

#### 4.2.1. Experimental Results with Changing α

**Figure 4.**
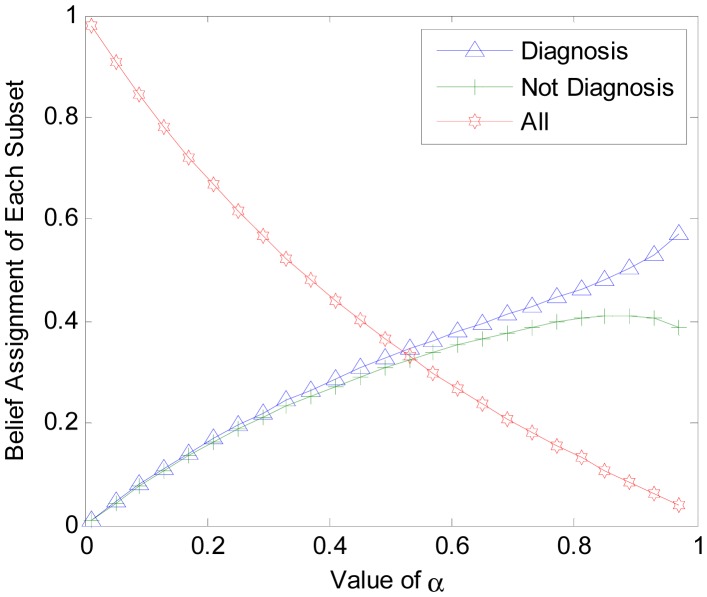
BBAs obtained with different α.

**Figure 5.**
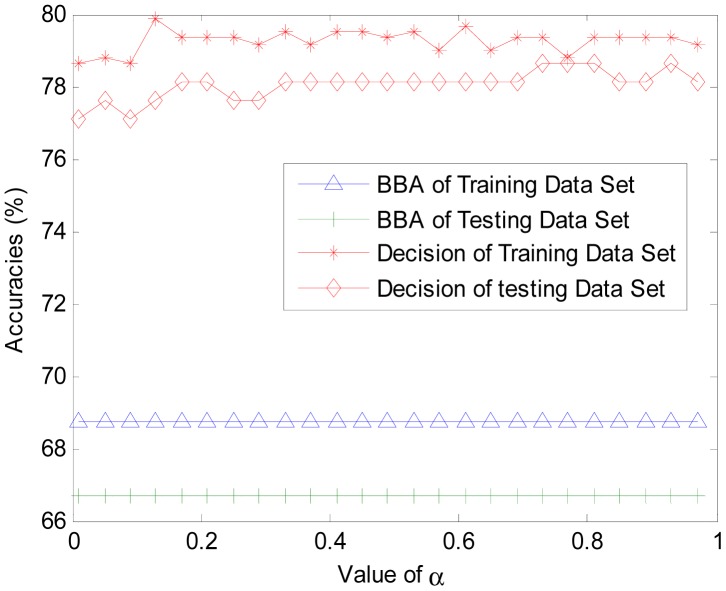
Accuracies with different α.

To get a better understanding of the proposed algorithm, additional experiments are conducted. In another trial, we set different value of α to find out its influences on BBA constructing and final result accuracy. The object is selected randomly from the test data set. Three classes of the power set are {diagnosis, Not diagnosis , All}, where ‘All’ denotes the compound set. Parameter α is set monotonically, increasing from 0 to 1. The values are used to calculate the corresponding BBAs. The BBA obtained with different α is shown in Figure 4. The accuracies of final unified BBA and final decision are shown in Figure 5.

In Figure 4, the BBA is obtained from the same object belonging to the diabetes diagnosis class. In Figure 5, the accuracy rate of BBA and decisions are both determined based on the training data set and testing data. From Figures 4 and 5, we can make the following conclusions:

(1) Parameter α will change the belief assignments of each subset in 2^Ω^. When α is closer to 0, m (Ω) ≈ 1 and the belief assignments of singleton class are close to 0. With an increasing α,*m*(Ω) decreases to a very low level while the belief assignments of singleton class increases to high levels. The gap between them will gradually diminish. However, it is strongly advised to set α > 0.7 to get a high differentiation degree for the BBA.
(2) Parameter α has no influence on the average accuracy of the BBA and decisions. In Figure 4, the BBA is larger for ‘Diagnosis’ compared to ‘Not Diagnosis”, regardless of the value of α. In Figure 5, the accuracies of unified BBAs in training data set and testing data set are 68.7 and 66.7, respectively. The accuracies of final decision results in training data set and testing data set are about 79% and 78%, respectively. Note that the decision accuracy fluctuation is caused by the instability of ELM. The stable accuracy rates illustrate that α has no influence on the accuracy of the BBA and decision accuracy.

#### 4.2.2. Experimental Results of Accuracies

Many algorithms and methods, such as BP neural network [28], Support Vector Machine (SVM) [29], ELM [22–24], and others, can use the database to get the classification results. To obtain a clear picture of the performance, we compare different algorithms, including BPNN, SVM, ELM, evidential data fusion with Pignistic transfer method (DSET-P) and the proposed DSET-E.

In this test, the parameter *C* of SVM algorithm is set at 10, and its accuracy results are obtained with 317 support vectors in average. All hidden nodes of the BPNN and ELM are 20. The DSET-P and DSET-ELM use the same process of calculating the unified BBA, thus their final BBAs are the same. α is set at 1 and the BBA function uses only the Mahalanobis distance because we have found that it has a high accuracy in constructing masses. In DSET -P, the unified BBA is converted to probability using the Pignistic transferring method in [19]. While in DSET-E, unified BBA is the input of a trained ELM, which is used to transfer the BBA to results and make decisions.

As shown in Table 2, all accuracies are calculated by the average accuracy results of repeating 100 times. From Table 2, we have the following observations:

(1) The proposed DSET-E algorithm performed well in classifying problems. Compared with other algorithms, the proposed DSET-E algorithm obtains a testing rate of 78.14%, outperforming other methods, though the improvement is not sufficiently distinctive. Compared with the DSET-P method, the accuracy increases from 66.67% to 78.14%, which is sufficient to prove that the whole algorithm is reasonable and effective.
(2) The traditional Pignistic transferring method is not a desirable algorithm in evidential data fusion, especially in situations with high complex and nonlinear data sources. The accuracy of DSET-P is 66.7%, which is much lower compared to the accuracies of other methods. Actually, in this problem, the highly complex source data are difficult to distinguish, and the final unified BBA has a low accuracy when calculated by Equation (30). The final decision made by Pignistic probability transferring model has the same accuracy as the unified BBA, which is 66.67%. With the same final unified BBA, the DSET-E decision accuracy rate of the test objects increases by 11.74%.
(3) The belief assignments of the compound classes are also important in decision-making. In traditional Pignistic probability transferring model, the belief assignments of a compound class are carved up by proportion, which makes no difference in decision-making. Apparently, it is not suitable for all conditions. The belief assignments of a compound class show uncertainty, making it difficult to decide to which class it should belong. It should be allocated to other singleton classes according to the reality situations.

### 4.3. Experiment on Vehicle Type Classification Data Set

In this experiment, a data set for vehicle type classification (the data set can be downloaded at [30]) is used to test the proposed algorithm. In the test, 23 wireless distributed sensor nodes are used to classify the types of the vehicles. When a vehicle is passing by, the nearby sensor nodes are able to record the signals in three modalities: acoustic, seismic and infrared. We use the recorded acoustic and seismic signals to classify two possible vehicles: Assault Amphibian Vehicle (AAV) and DragonWagon (DW). Before classification, feature vectors must be extracted from the raw signals. A detailed introduction of the feature extraction method can be found in [31].

The experiment includes two parts: part one is the classification based on the whole data set. In this scenario, universal classification algorithms can be directly used and their classification results will be presented; part two is the classification conducted in a distributed multisensor data fusion way. Experimental results of local classification and data fusion will also be presented in the following sections.

In the first test, five classification methods are used, including k-NN, ELM, SVM, DSET-P and DSET-E. The sample set is consisted by 535 feature vectors, which are randomly selected from the provided whole feature data set. Among the sample set, 277 feature vectors belong to vehicle AAV, the rest are DW. The valid data set has 236 feature vectors, which are also randomly selected from the whole feature data set. The classification results are given in the following Table 3.

As shown in Table 1, five classification algorithms are used for local classification. The parameter *k* in *k*-NN method, hidden nodes number of ELM and parameter *C* of SVM are set as 15, 100 and 1, respectively. The mass construction used in DSET-P and DSET-E is the method proposed in Section 3.2 and parameter *α* is set as 0.85. From Table 3, we can conclude that the proposed DSET-E has a more reliable result than other methods, which is consistent with the results of Table 2.

Then we conducted the task in a multisensor data fusion scenario, in which each sensor node has its own sample data set collected by itself. Since the energy and bandwidth of wireless sensors are strictly limited, uploading the raw data to the sink node is unpractical. Therefore, a local classification in the sensor node needs to be conducted and then these local results are uploaded to the fusion center for final decision by data fusion algorithms. Except for DSET-P and DSET-E, the algorithms used in Table 3 cannot be used for classification. The local classification accuracies the final data fusion accuracies are shown in Tables 2 and 3, respectively.

As shown in Tables 4 and 5, there are 11 sensor nodes used for the collaborative data fusion task. Both test set have 1177 vector samples, in which 615 vectors belong to AAV, the other 562 vectors are DW. From Tables 3 to Table 5, it can be concluded that the proposed method has good performance of in multisensor data fusion applications, because it is able to get reliable and robust results. In Tables 2 and 3, the accuracies of DSET-E are always higher than the accuracies of other classification methods. In Table 4, the average classification accuracies of AAV and DW are 68.13% and 59.83%, respectively. However, the fusion results are greatly improved by both the DSET-P and DSET-E methods. The final average accuracy of DSET-E is 75.32%, which increased by 2.52% compared to DSET-P. And also, the average accuracy of DSET-E is close to the results of DSET-E in Table 3, which equals to 76.62%. These results also show that the proposed BBA constructing function is reasonable and effective for the DSET based data fusion framework.

To conclude the experimental section, the three tests prove that the proposed mass constructing and decision making method is reasonable and practical. The classification results with other universal classification methods (*i.e.*, *k*-NN, BPNN, ELM and SVM) prove that the proposed method is able to obtain robust and reliable results. The experiment on vehicle type classification demonstrates that the proposed method has high performance in multisensor data fusion applications, but not only in practice for conventional problems. Therefore, the proposed model is robust and reliable in mutisensor data fusion.

## 5. Discussion and Conclusions

In conclusion, this paper proposed a systematic multisensor data fusion model to obtain robust and high-precision fusion results. DSET is used to provide a flexible way to combine multiple information sources into a unified one, and ELM is applied to make decisions. The combination of the two theories achieves a greater capacity in multisensor data fusion. Compared with the existing methods, the proposed framework gives more flexibility and rationality in constructing reasonable BBAs from data sources. Additionally, the framework is able to make decisions according to the actual situation. Moreover, it is stable and easy to implement. With adequate training samples, the algorithm is able to reason and learn and make decisions in a coherent process. The drawback is that it needs to be trained and the computation complexity is greater than that of traditional DSET-P, though ELM is ‘extremely’ fast in ANNs. However, it should be clear that the proposed method is not intended to achieve great improvement over other classification algorithms, but rather it is aimed at building a robust and reliable data fusion model practical for multisensor applications. Thus, the accuracy improvement is not the key point of the proposed model. Though the improvement of training accuracy and testing accuracy is not significant, the results still prove that the proposed is robust and reliable.

It is necessary to emphasize that in Section 3.3, ‘minority is subordinate to the majority’ underlies the synthetizing algorithm, which is useful when the performance of the adopted BBA functions or experts are unknown to us. It can be modified as ‘outstanding is preferred’, which means the weights of the BBA functions or experts are assigned according to their own accuracies. In Section 3.5, the activation function is the ‘radbas’ function. Other function types are also feasible. In Section 4.2, the experimental results indicate that a decision-making method exists after the combinational step, although it is not the Pignistic transferring method. If we could improve the conventional Pignistic transfer method, DSET could be greatly promoted in real applications. Future work may involve the following: (1) discovering a new decision-making method to get rid of the low accuracy limitation of the existing Pignistic methods; (2) developing a DSET-embedded ELM that is able to deal with pattern recognition or classification problems; and (3) exploring more inherent laws in the transducer mechanism and probability.

## Figures and Tables

**Table 1.** Accuracies of the BBAs obtained for the IRIS data (%).

	**SL**	**SW**	**PL**	**PW**	**SL, SW**	**PL, PW**	**All**
Eu-BBA	73.33	58.33	96.67	96.67	83.33	96.67	98.33
Mahal-BBA	80.00	58.33	96.67	96.67	86.67	96.67	98.33
Ch-BBA	73.33	58.33	96.67	96.67	83.33	96.67	95.00
Ma-BBA	73.33	58.33	96.67	96.67	81.67	95.00	96.67
Syn-BBA	73.33	58.33	96.67	96.67	86.67	96.67	96.67

**Table 2.** Accuracy comparison for DIABETES data set (average, %).

**Algorithms**	**Training Accuracies**	**Testing Accuracies**
DSET-E	79.39	78.14
DSET-P	-	66.67
ELM	78.68	77.57
SVM	78.76	77.31
BPNN	86.63	74.73

**Table 3.** Classification results of different methods.

**Algorithm**	**AAV**	**DW**	**Average**
*k*-NN	70.11	70.57	70.32
ELM	73.90	78.28	76.06
SVM	62.51	**81.55**	71.64
DSET-P	73.54	76.22	74.93
DSET-E	**76.01**	76.79	**76.62**

**Table 4.** Local classification accuracy of each sensor node (%).

**Node**	**1**	**2**	**3**	**4**	**5**	**6**	**7**	**8**	**9**	**10**	**11**	**Average**
AAV	56.42	53.04	85.35	65.31	75.29	85.43	62.52	63.52	70.24	66.57	65.73	68.13
DW	72.12	43.37	45.67	62.81	48.70	48.57	75.80	64.54	63.38	62.55	70.64	59.83

**Table 5.** Final data fusion accuracies (average, %).

	**AAV**	**DW**	**Average**
DSET-P	79.84	65.50	72.80
DSET-E	82.41	67.57	75.32

## References

[1] Khaleghi B., Khamis A., Karray F.O., Razavi S.N. (2011). Multisensor data fusion: A review of the state-of-the-art. Inf. Fusion.

[2] Al Momani B., Morrow P., McClean S. (2011). Fusion of Elevation Data into Satellite Image Classification Using Refined Production Rules. Image Analysis and Recognition.

[3] Lelandais B., Gardin I., Mouchard L., Vera P., Ruan S. (2012). Using Belief Function Theory to Deal with Uncertainties and Imprecisions in Image Processing. Belief Functions: Theory and Applications.

[4] Deng Y., Chan F.T.S. (2011). A new fuzzy Dempster MCDM method and its application in supplier selection. Expert Syst. Appl..

[5] Guerriero M., Svensson L., Willett P. (2010). Bayesian data fusion for distributed target detection in sensor networks. IEEE Trans. Signal Process..

[6] Du P., Liu S., Xia J., Zhao Y. (2013). Information fusion techniques for change detection from multi-temporal remote sensing images. Inf. Fusion.

[7] Zimmermann H J. (2001). Fuzzy Set Theory—And Its Applications.

[8] Qian Y., Li S., Liang J., Shi Z., Wang F. (2014). Pessimistic rough set based decisions: A multigranulation fusion strategy. Inf. Sci..

[9] Shafer G. (1976). A Mathematical Theory of Evidence.

[10] Yager R., Fedrizzi M., Kacprzyk J. (1994). Advances in the Dempster-Shafer Theory of Evidence.

[11] Zhang Z., Liu T., Zhang W. (2014). Novel Paradigm for Constructing Masses in Dempster-Shafer Evidence Theory for Wireless Sensor Network's Multisource Data Fusion. Sensors.

[12] Chakeri A., Nekooimehr I., Hall L.O. Dempster-Shafer Theory of Evidence in Single Pass Fuzzy C Means.

[13] Ben Chaabane S., Fnaiech F., Sayadi M., Brassart E. Estimation of the Mass Function in the Dempster-Shafer's Evidence Theory Using Automatic Thresholding for Color Image Segmentation.

[14] Zhu H., Basir O. A Scheme for Constructing Evidence Structures in Dempster-Shafer Evidence Theory for Data Fusion.

[15] Basir O., Karray F., Zhu H. (2005). Connectionist-based Dempster-Shafer evidential reasoning for data fusion. IEEE Trans. Neural Netw..

[16] Zhu H., Basir O. (2006). A novel fuzzy evidential reasoning paradigm for data fusion with applications in image processing. Soft Comput..

[17] Smets P., Kennes R. (1994). The transferable belief model. Artif. Intell..

[18] Smets P. (2005). Decision making in the TBM: The necessity of the pignistic transformation. Int. J. Approx. Reason..

[19] Sudano J. Pignistic Probability Transforms for Mixes of Low- and High-Probability Events. http://www.incose.org/delvalley/FUSION2001.pdf.

[20] Chen S., Deng Y., Wu J. (2013). Fuzzy sensor fusion based on evidence theory and its application. Appl. Artif. Intell..

[21] Jousselme A.-L., Grenier D., Bossé E. (2001). A new distance between two bodies of evidence. Inf. Fusion.

[22] Huang G.B., Zhou H., Ding X., Zhang R. (2012). Extreme learning machine for regression and multiclass classification. IEEE Trans. Syst. Man Cybern. Part B Cybern..

[23] Zouhal L.M., Denœux T. (1998). An evidence-theoretic k-NN rule with parameter optimization. IEEE Trans. Syst. Man Cybern. Part C Appl. Rev..

[24] Huang G.B., Zhu Q.Y., Siew C.K. (2006). Extreme learning machine: Theory and applications. Neurocomputing.

[25] Huang G.B., Zhu Q.Y., Siew C.K. Extreme Learning Machine: A New Learning Scheme of Feedforward Neural Networks.

[26] Fish R.A. (1936). The use of multiple measurements in taxonomic problems. Ann. Eugen..

[27] Pima Indians Diabetes Data Set. http://archive.ics.uci.edu/ml/datasets/Pima+Indians+Diabetes.

[28] Gupta C.N., Palaniappan R., Swaminathan S., Krishnan S.M. (2007). Neural network classification of homomorphic segmented heart sounds. Appl. Soft Comput..

[29] Bazi Y., Melgani F. (2006). Toward an optimal SVM classification system for hyperspectral remote sensing images. IEEE Trans. Geosci. Remote Sens..

[30] Matlab Toolboxes. http://www.ecs.umass.edu/~mduarte/Software.html.

[31] Duarte M.F., Hen H.Y. (2004). Vehicle classification in distributed sensor networks. J. Parallel Distrib. Comput..

